# Supplementary figures and images for: Optimizing an eDNA protocol for estuarine environments: Balancing sensitivity, cost and time
Source: PLoS One. 2020 May 21;15(5):e0233522. doi: 10.1371/journal.pone.0233522 (PMC7241769; doi:10.1371/journal.pone.0233522)

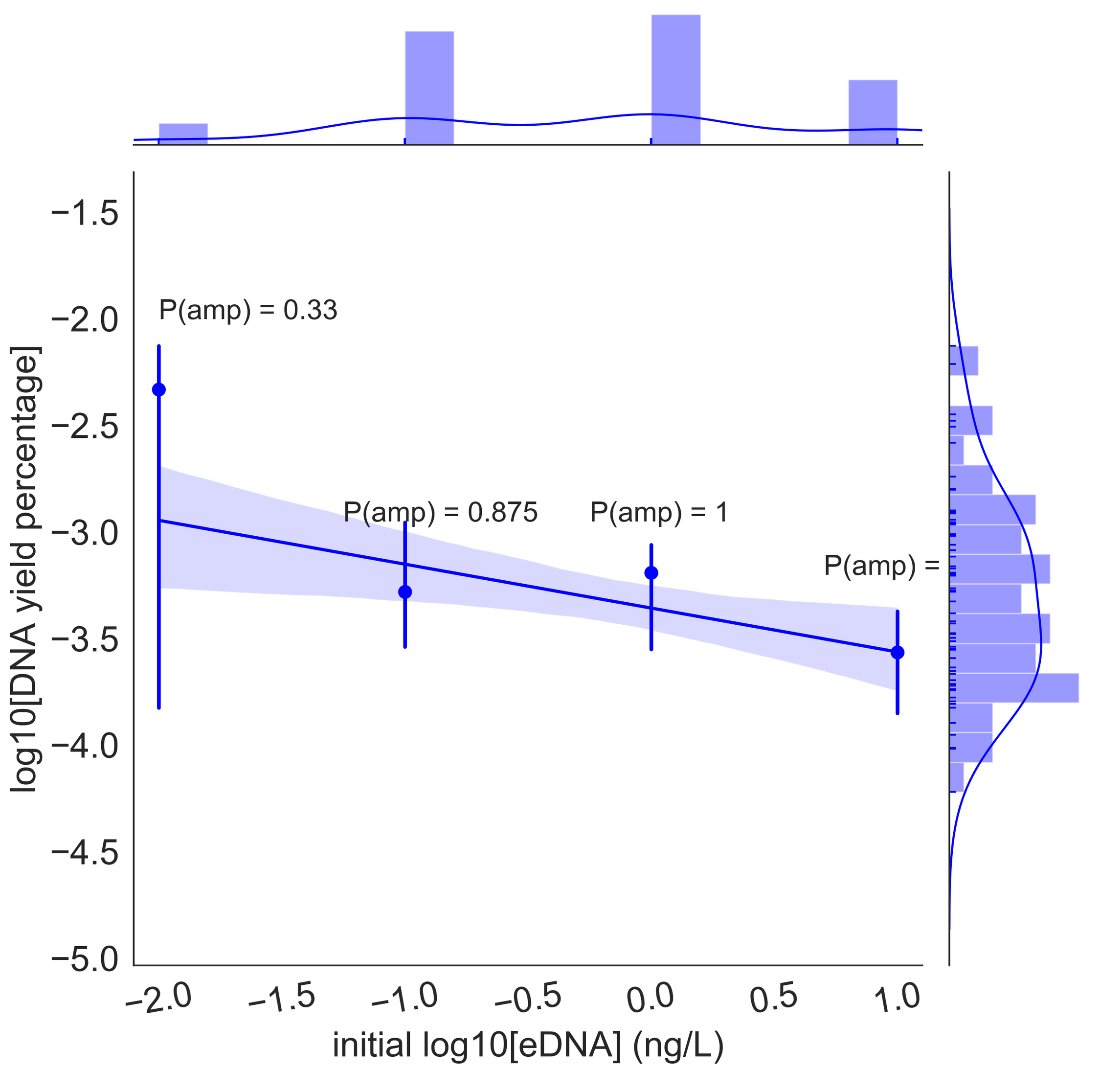

Supplement: S1 Fig — Blue dots—median value; vertical lines—95% CI; horizontal line—linear regression between protocol DNA yield and input DNA, with both axes being represented in log10 scale. P(amp)—probability of amplification. (TIF) [file pone.0233522.s001.tif]

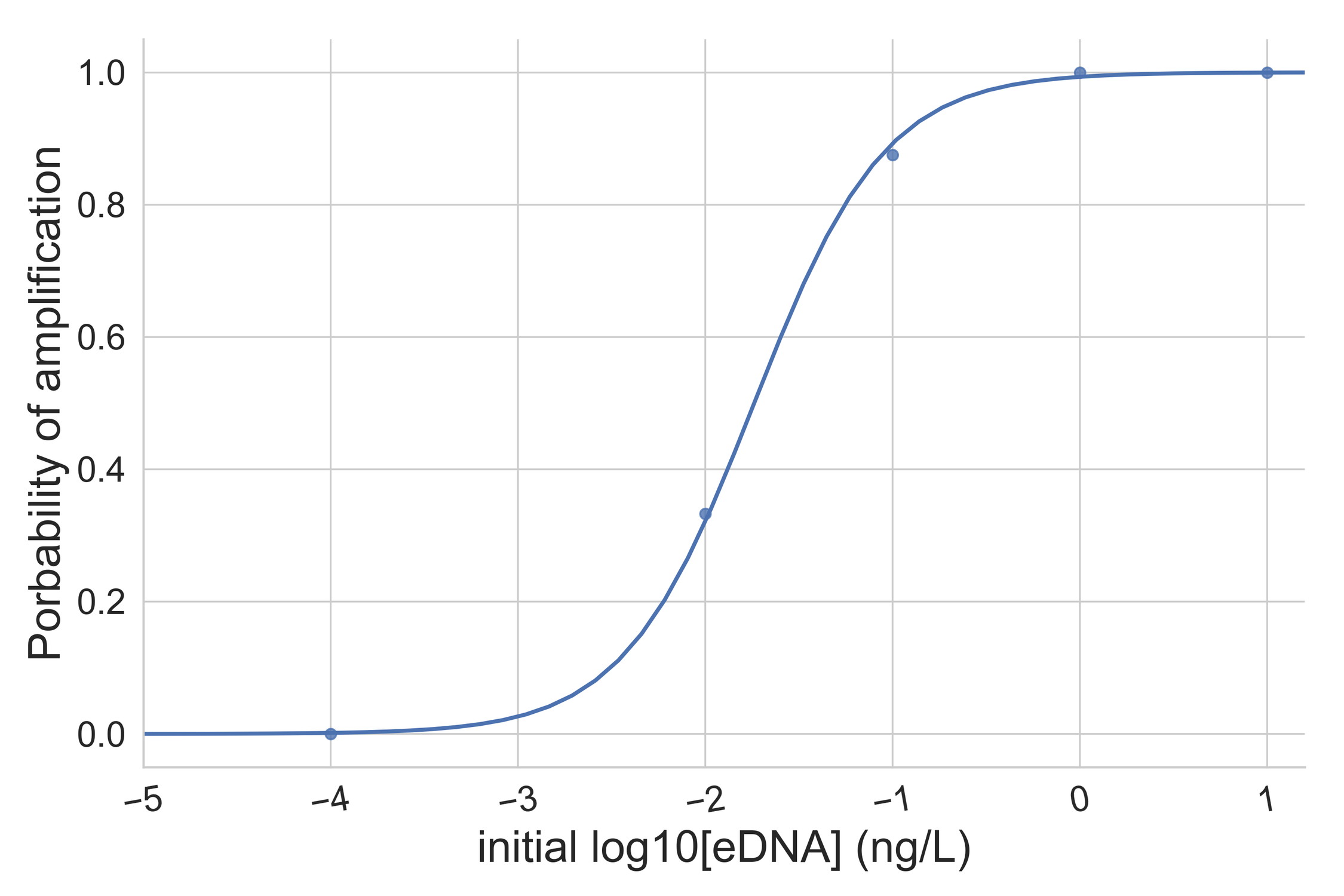

Supplement: S2 Fig — Dots—probability of amplification from DNA spiking experiment; line—logistic fit to data points. (TIF) [file pone.0233522.s002.tif]
