# Supplementary material for: Optimizing an eDNA protocol for estuarine environments: Balancing sensitivity, cost and time
Source: PLoS One. 2020 May 21;15(5):e0233522. doi: 10.1371/journal.pone.0233522 (PMC7241769; doi:10.1371/journal.pone.0233522)
Supplement: Attachment — Submitted filename: rebutal letter optimization paper.pdf [file pone.0233522.s003.pdf]

Rebuttal letter

We thank the editor and the two reviewers for their comments on our manuscript. Below is our response to each point raised by the academic editor and reviewers. We hope that we satisfyingly addressed them and that the manuscript will be now suited for publication.

Sincerely,

On behalf of all authors,

Thiago Sanches

Academic editor:

1. Please ensure that your manuscript meets PLOS ONE's style requirements, including those for file naming.

File naming was edited to comply with the style requirements. We hopefully have no divergences from the style requirements now.

2. Thank you for including your ethics statement:

'We sampled water in accordance with the University of California Davis Institutional Animal Care and Use Committee (USDA registration: 93-R-0433, PHS Animal Assurance A3433-01) under the protocol number #20608.'

a. Please amend your current ethics statement to confirm that your named ethics committee specifically approved this study.

We amended the ethics statements to show that the study was approved by UCD IACUC on line 124-126:

"Holding juvenile Chinook salmon in captivity to sample water for this study was approved by the University of California Davis Institutional Animal Care and Use Committee (USDA registration: 93-R-0433, PHS Animal Assurance A3433-01) under the protocol number #20608."

b. Once you have amended this statement in the Methods section of the manuscript, please add the same text to the "Ethics Statement" field of the submission form (via "Edit Submission").

The ethics statement was also amended in the submission form.

3. Please amend either the title on the online submission form (via Edit Submission) or the title in the manuscript so that they are identical.

The title was also amended in the submission form.

4. Please ensure that you refer to Figure 9 in your text as, if accepted, production will need this reference to link the reader to the figure

Figure 9 is now referenced on line 453

5. We note you have included a table to which you do not refer in the text of your manuscript. Please ensure that you refer to Table 1 in your text; if accepted, production will need this reference to link the reader to the Table.

Table 1 is now referenced on line 265

Additional Editor Comments (if provided):

I got the reviewers' comments for the manuscript. The both reviewers are positive for the publication but found some revision points, e.g., the structure of the manuscript needs to be reorganized, the results were not fully discussed, and sufficient explanations are lacking in some paragraphs. I totally share the comments and recommend to revise for reconsidering the publication.

We modified the manuscript to address the points made by the editor and the reviewers. We agreed with the comments in all accounts. We believe that the manuscript is now more readable, more informative, and its conclusions more useful to the public.

Reviewer #1

The structure of the manuscript

The main body of the manuscript should be reorganized.

(1) The methods section should come before the results, as the focus of this study is to evaluate the effects of each step over the eDNA experimental procedure on the results. In other words, the method is pretty important for this study.

The order of the sections was rearranged and now the methods section is after the introduction.

(2) Subsectioning in the Introduction is not necessary.

Sub Sectioning in the introduction was removed and the flow of the reading in the introduction section was ameliorated

(3) Two subsections (estuary waters and Chinook salmon) can be moved to the materials and methods section if they are just a case/example. Or, if there are any special biological reasons to employ them in this study, how this study contributes to address those biological questions should be discussed in the discussion part.

We opted to shorten the Chinook salmon and estuarine waters section and keep that information in the introduction without the subsections. We believe in this case, as eDNA is

highly dependent on the tested environment. A brief mention of our system in the introduction would improve readability.

(4) The results and discussions should be separated. The interpretations of the results of this study are not very very easy, so the readers can understand the results more easily if the authors provide more detailed explanations on each result. Then, the authors can make sufficient discussions by combining all the results.

We separated the results from the discussion while improving the explanation of the results. We opted to provide the discussion section without subheading while including comparisons of our results to the literature.

(5) The current subsection titles in the results and discussion are too long. They should be renamed by making the discussing points clear.

We shortened the subsection titles while also clarifying the goal of each subsection.

Abstract

Methods and results are not mentioned. They should be included in Abstract.

We amended the abstract to include methods and results in a concise manner on lines 20-28

Introduction

For each of the four steps that the authors determined, they should be explained fairly about what a problem can be, what should be examined and how those problems are addressed.

We added information about the specifics of each step, mentioning the problems in each step and how to address them.

Results

Filter type (Fig. 1); the authors did not test some types of filters that are also commonly used in eDNA studies such as nylon and polyvinylidene difluoride (PVDF) filters. The authors should explain why they chose paper filter, glass fibre and nitrocellulose.

The choice of filter materials is now explained on lines 396-399. Tested filter materials were chosen as nitrocellulose has, in general, the highest DNA retention rate in the literature, while glass fiber is currently the most common filter material while Whatman paper was tested as an experimental material which main advantage is the availability and its cost being a fraction of other filter materials.

Filtration time (Fig. 3); the authors did not present the pore sizes of the filters examined. Thus, the relevance of the results could not be evaluated.

Pore sizes in Figure 3 are now reported and the reasoning for the chosen pore sizes is described on line 400-401.

DNA extraction; the dipstick direct to qPCR approach seems to be in contradiction with the fact that the authors made account of PCR inhibitor removal. The explanation on inclusion of this approach should be briefly provided.

An explanation for the observation and reasoning for including this approach is now described on line 239, 247-251.

The starting point of the flow (Fig. 7; does the interest species occupy a high percentage of the sampled sites?) seems a bit special situation in practice. The researchers who employ the eDNA technique generally want/need to estimate the distribution and/or abundance of the species of interest or species community, so it is as if that starting question is the final goals of a study. In addition, the term of a high percentage is unclear (high abundance, density, biomass …?). Guessing from the title of this manuscript, either cost or time may be probably more adequate.

We addressed this question by rewording the figure, changing from species abundance to confidence in negative tests (“are false negatives a major concern”). We also noticed that the figure explanation in the text was subpar and we addressed it by justifying every section of the decision tree. Therefore we expect the figure to be more legible and contain more useful information for our target audience. Changes can be seen on lines 455-470

Discussions

As a large number of technical studies for eDNA analyses have been published, there should be many aspects to be discussed, which are definitely lacking in the present manuscript. The authors apparently aimed at developing a kind of universal guideline to make a decision on choosing an eDNA analysis protocol, so the broad discussions including for example comparisons with previous studies are necessary, which also increases the scientific value of this study.

We added comparative information with other studies and made more clear the novel observations of our study compared to others. Changes are described on lines 404-414

Reviewer #2:

Authors aimed to optimize environmental DNA pipeline, filtration, DNA extraction, PCR inhibitor removal and DNA amplification. The results can be useful not only for the eDNA users but also the person who is considering to start eDNA monitoring. Particularly, evaluating specific costs and bifurcation chart are the novelty of this article. However, eDNA studies are updating day by day, we need ongoing updates of this conclusion. And you should explain what kinds of PCR inhibitor were included in your samples and excluded from your samples.

We added the types of PCR inhibitors we would expect in our samples on lines 97-98. We added a comment about the fast evolution of the field and how our data analysis and study design might still be useful even if our methods get obsolete lines 404-414.
